# Virtual Learning Environment of the Brazilian Health System (AVASUS): Efficiency of Results, Impacts, and Contributions

**DOI:** 10.3389/fmed.2022.896208

**Published:** 2022-06-02

**Authors:** Ricardo A. M. Valentim, Carlos A. P. de Oliveira, Eloiza S. G. Oliveira, Eduardo L. Ribeiro, Soneide M. da Costa, Ione R. D. Morais, Felipe R. dos S. Fernandes, Alexandre R. Caitano, Cristine M. G. Gusmão, Aliete Cunha-Oliveira, Maria C. F. D. Rêgo, Karilany D. Coutinho, Daniele M. S. Barros, Ricardo B. Ceccim

**Affiliations:** ^1^Laboratory for Technological Innovation in Health, Federal University of Rio Grande Do Norte, Natal, Brazil; ^2^Multidisciplinary Institute of Human Development With Technologies, State University of Rio de Janeiro, Rio de Janeiro, Brazil; ^3^Department of Distance Education of the Federal University of Rio Grande Do Norte, Natal, Brazil; ^4^Department of Biomedical Engineering, Federal University of Pernambuco, Recife, Brazil; ^5^Health Sciences Research Unit: Nursing (UICISA: E) and Nursing School of Coimbra (ESEnfC), Portugal and CEIS-20 da Universidade de Coimbra, Coimbra, Portugal; ^6^Graduate Program in Education, Federal University of Rio Grande Do Sul, Porto Alegre, Brazil

**Keywords:** AVASUS, lifelong learning in health, permanent education in health, coverage, scalability

## Abstract

The Virtual Learning Environment of the Brazilian Health System (AVASUS) is a free and open distance education platform of the Ministry of Health (MS). AVASUS is a scalable virtual learning environment that has surpassed 800,000 users, 2 million enrollments, and 310 courses in its catalog. The objective of this paper was to assess the impacts of the educational offerings on health services and AVASUS course participants' professional practice. This study analyzed data from AVASUS, the Brazilian National Registry of Health Care Facilities (CNES), the Brazilian Occupational Classification (CBO), and a questionnaire applied to 720-course participants from five regions of Brazil. After acquiring and extracting data, computational methods were used for the evaluation process. Only the responses of 462 participants were considered for data analysis, as they had a formal link to CNES. The results showed that respondents recommended 76.2% of AVASUS courses to peers. Accordingly, the quality of educational offerings motivated 81.3% of such recommendations. In addition, 75.6% of course participants who answered the questionnaire also indicated that AVASUS course contents contribute to enhancing existing health services in the health facilities where they work. Finally, 24.6% of all responses mentioned that courses available in AVASUS were essential in offering new health services in such facilities.

## 1. Introduction

Since the Brazilian Health System (SUS, for its acronym in Portuguese) was structured in the 1980s, health professional training has been one of its cornerstones. In addition, SUS structure is marked by a robust decentralization of the services offered. It, therefore, resulted in greater access of the population to Primary Health Care (PHC) through programs like “Family Health Strategy.” And the expansion of the population's access to medium- and high-complexity services was another critical factor (1, 2).

As SUS have expanded, the demand for a large health care workforce has increased (3). In 2020, according to the Brazilian National Registry of Health Care Facilities (CNES), Brazil had 305,765 health facilities (http://cnes.datasus.gov.br/), with a workforce of nearly 6,6 million public health workers.

There are many difficulties yet to be addressed in terms of health training, education, and daily demands of SUS. Faced with the fact that healthcare work has its specificities, health workers rely on articulating different sorts of knowledge based on scientific, instrumental, and technological grounds in their decision-making process. In such a process, continuing education comes forth as a pivotal instrument (4, 5). What is distinctive about continuing education in health is that it promotes education focused on teamwork and the comprehensiveness of care (6). In addition, emphasis is given to the possibility for health practitioners to learn and teach in their day-to-day work (5, 7–9).

In Brazil, the concept of “health professional education” denotes the construction of knowledge aimed to enhance work through the lens of health promotion in the national territory. Furthermore, the demand for continuous health education to keep professionals' knowledge current for their healthcare practices has been thoroughly discussed (10). Therefore, SUS provide fertile ground for applying technological solutions that enhance the quality of services delivered by the health system.

On October 14, 2015, the Virtual Learning Environment of the Brazilian Health System (AVASUS, https://avasus.ufrn.br/) was launched. It stemmed from a partnership between the Ministry of Health (MoH) and the Federal University of Rio Grande do Norte (UFRN). At UFRN, the Laboratory for Technological Innovation in Health (LAIS) and the Secretariat of Distance Education (SEDIS) developed such a virtual learning environment.

The purpose of AVASUS is to contribute to lifelong learning in health by providing scientifically sound information and resources to build a skilled and qualified healthcare workforce. This improves their ability to share knowledge at their workplace and incorporate it into their decision-making processes (11, 12). That said, its application is motivated by the demand for qualifying health professionals who work at the primary, secondary, and tertiary levels of health care.

This study analyzed data from the following sources: (i) AVASUS, (ii) CNES, (iii) the Brazilian Occupational Classification (CBO), and (iv) a questionnaire applied to 720-course participants. In this perspective, our objective was to assess the impact of the educational offerings on health services and AVASUS course participants' professional practice. In addition, we sought to elucidate the following guiding research questions:

- Do open and flexible distance learning courses foster the sharing of knowledge in the workplace?
- Can open and flexible distance learning courses meet the current demands of healthcare services?
- Do open and flexible distance learning courses enable innovative healthcare services or bolster existing ones?

### 1.1. Virtual Learning Environment of the Brazilian Health System (AVASUS)

AVASUS is an open and free virtual learning environment commissioned by the Brazilian Ministry of Health (MoH) to qualify healthcare professionals, students in the field of health, and the general public on healthcare (13). Courses, or educational modules, are developed through partnerships between higher education institutions and other institutions within the health sector (12, 14).

The platform is integrated into an educational ecosystem of the various MoH platforms, such as the Evidence-Based Health Portal (EBHP), Health Practices Community, and Telehealth Program. As part of the efforts of the MoH, AVASUS's mission is to promote accessible and integrated health knowledge, playing a pivotal role in continuing education (15).

Currently, AVASUS has 800,000 users, more than 2 million enrollments and includes 310 courses (https://avasus.ufrn.br/local/avasplugin/dashboard/transparency.php). Since its early stages, the platform has been deemed a valuable communication and learning tool for health workers and the population in general (16).

AVASUS has emerged as a swell virtual learning environment for the SUS workforce and society throughout three major public health crises in Brazil (see Table 1). In 2015, for instance, an unexpected surge in the number of cases of live births with microcephaly–a congenital malformation characterized by a reduced head circumference for gestational age, along with changes in the central nervous system–was observed in Brazil (17, 18).

**Table 1:** Total number of attendees during three Brazilian public health crises.

	**Regions of Brazil**					**Total**
**Public health emergency**	**North**	**Northeast**	**South**	**Southeast**	**Midwest**	
Syphilis	12,349	47,479	22,265	50,328	13,952	146,373
COVID-19	15,374	106,359	39,059	91,856	36,251	288,899
Zika Virus and Microcephaly	5,759	27,430	11,816	27,769	9,012	81,786

Some of the courses offered are as follows: Early Stimulation, Qualification in Eye Screening, Zika: Clinical Approach in PHC, Emerging Diseases (Dengue, Zika Virus, Chikungunya, and others). Altogether, the courses sum up to 370 h of content, with 81,786 attendants distributed among the country's five regions. Table 1 depicts the number of attendees per region. Particularly noteworthy is the Southeast region, with 27,769 students enrolled in a course in such a learning pathway (19).

Learning pathways about syphilis should also be emphasized. In 2016, after the Syphilis epidemic was declared in Brazil, the Brazilian government developed a national plan with strategic actions to fight against the infection (20–23). Among these actions, the Education dimension stood out. Through AVASUS, a learning pathway with 49 courses was made available, which now has 146,373 participants (15).

The usage of AVASUS on the COVID-19 pandemic is another highlight (16). Some available courses on the topic are “Prenatal Care and Puerperium in Times of COVID-19”; “COVID-19: Protocol for Clinical Management of Coronavirus in Primary Health Care”; “Development of Vaccines and Therapies to Combat COVID-19”; and “COVID-19: Caring for the Elderly in Long-Term Care Institutions.” So far, this learning pathway related to COVID-19 has 288,899 attendants nationwide. Northeast Brazil holds the record of enrollments, with 106,359 participants. Such numbers reflect the relevance of AVASUS in the context of the Brazilian health system, as well as in promoting continuing education for health workers.

## 2. Materials and Methods

The development of the present study consisted of the following four steps: (i) Determining Survey Sample Size; (ii) Data Acquisition; (iii) Application of the questionnaire; (iv) Data Processing.

### 2.1. Determining Survey Sample Size

Sample definition was set based on the total number of enrollees in the AVASUS as of December 2018. By that year, overall enrollment in available courses was 647,144, whereby 115,984 enrollees had completed at least 70% of a given course. To obtain the minimum sample size necessary for estimating the variable of interest, it was considered that the population N ≥ 80,000 enrollments, i.e., the population of interest is about 64% of AVASUS course participants.

The equation below was to calculate the sample size:


(1) $$\begin{array}{r} n=\frac{NZ_{\frac{\alpha }{2}}^{2}p\left(1-2\right)}{Z_{\frac{\alpha }{2}}^{2^{p}}\left(1-p\right)+\left(N-1\right)e^{2}} \end{array}$$

where,

- *n*: minimum sample size
- *N*: population size
- α: significance level
- *p*: proportion of the population with the features of interest
- $Z_{\frac{\alpha }{2}}$: percentile of the normal distribution for α/2.

We considered α and e of 0.05 (5%), which is the maximum difference allowed between estimated values and true values; and, for the value of p, we attributed 0.5 (50%), corresponding to the worst scenario in terms of estimation, and thus provides the largest sample size value.

Finally, we found the sample size necessary to derive the results and the maximum permissible error (n = 383 questionnaire participants) according to the equation and the confidence interval adopted.

### 2.2. Data Acquisition

To build the dataset “survey_results.csv” (available in: Zenodo, see link in section 6) collected data from the following sources: (i) AVASUS, (2) the questionnaire survey administered to AVASUS users, CNES, and CBO. After compiling questionnaire data, a unique user identifier code was created for data anonymization, cross-reference, and collection from CNES and CBO, respectively. The purpose of extracting data from CNES was to collect user information related to occupation, e.g., CBO code, place of residence, and place of work.

### 2.3. Application of the Questionnaire

The questionnaire, our data collection instrument, was provided to all users who had completed a minimum of 70% of at least one course/module in AVASUS from November 16, 2018, to December 17, 2018 (the questionnaire is available at: Zenodo, see link in section 6). During this timeframe, 720 participants submitted their responses, of which 462 were healthcare workers. This means that the sample size is 20.62% above the minimum required. A committee of five experts who had doctoral degrees and at least ten years of experience in the field of education refined, evaluated, and validated the questionnaire before its administration. Out of the five experts, two work in health education and technological mediation, one has experience in education management and works at SEDIS, one works in health education and has vast experience in community health agent education, and one works with statistical analysis applied to education.

### 2.4. Data Processing

A 3-stage pipeline was executed in Python language for data processing, treatment, and record linkage. In the first stage, feature extraction, data from all sources were cross-referenced in order to create news user features, such as a description of profession and spatial distribution. In the second stage, feature selection, we selected the main features to carry out dataset anonymization and thus accomplish the aim of the present study.

As for the third stage, record linkage, the purpose was to carry out data comparison and linkage of the different databases (24). Using the CNES number found in the AVASUS records, a search on the CNES database was performed. This process enabled the identification of the place of work, the territory of professional practice, and the occupation. Then, the CBO code, also available in AVASUS, was searched for in CBO data. This process enabled the identification of participants' professions.

## 3. Results

**Figure 1:**
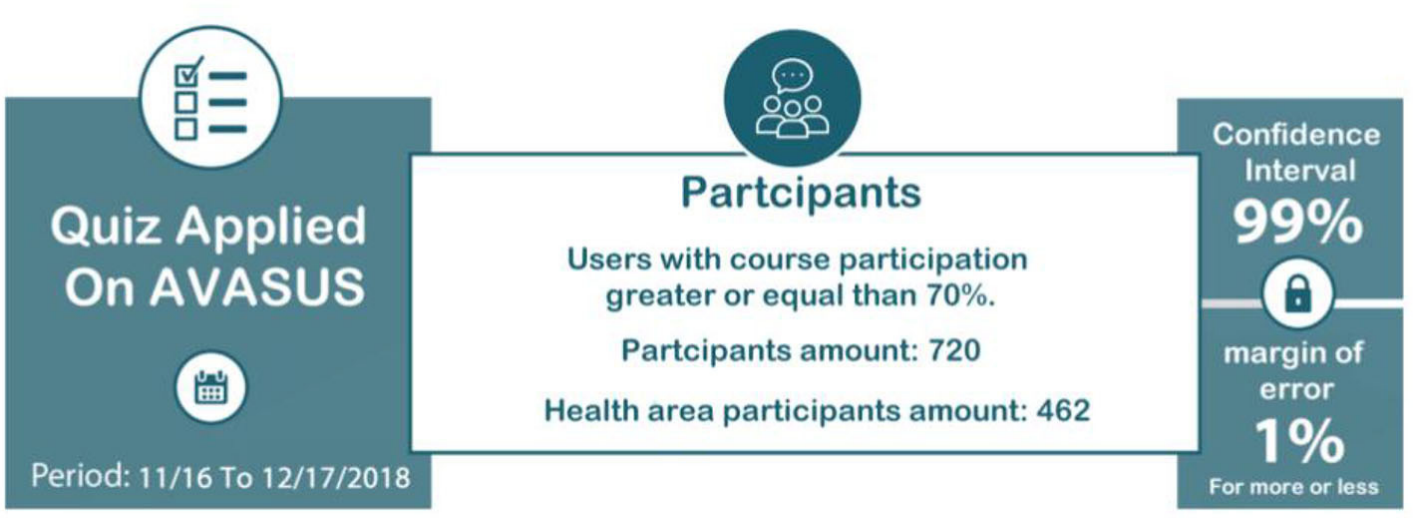
Description of data.

**Figure 2:**
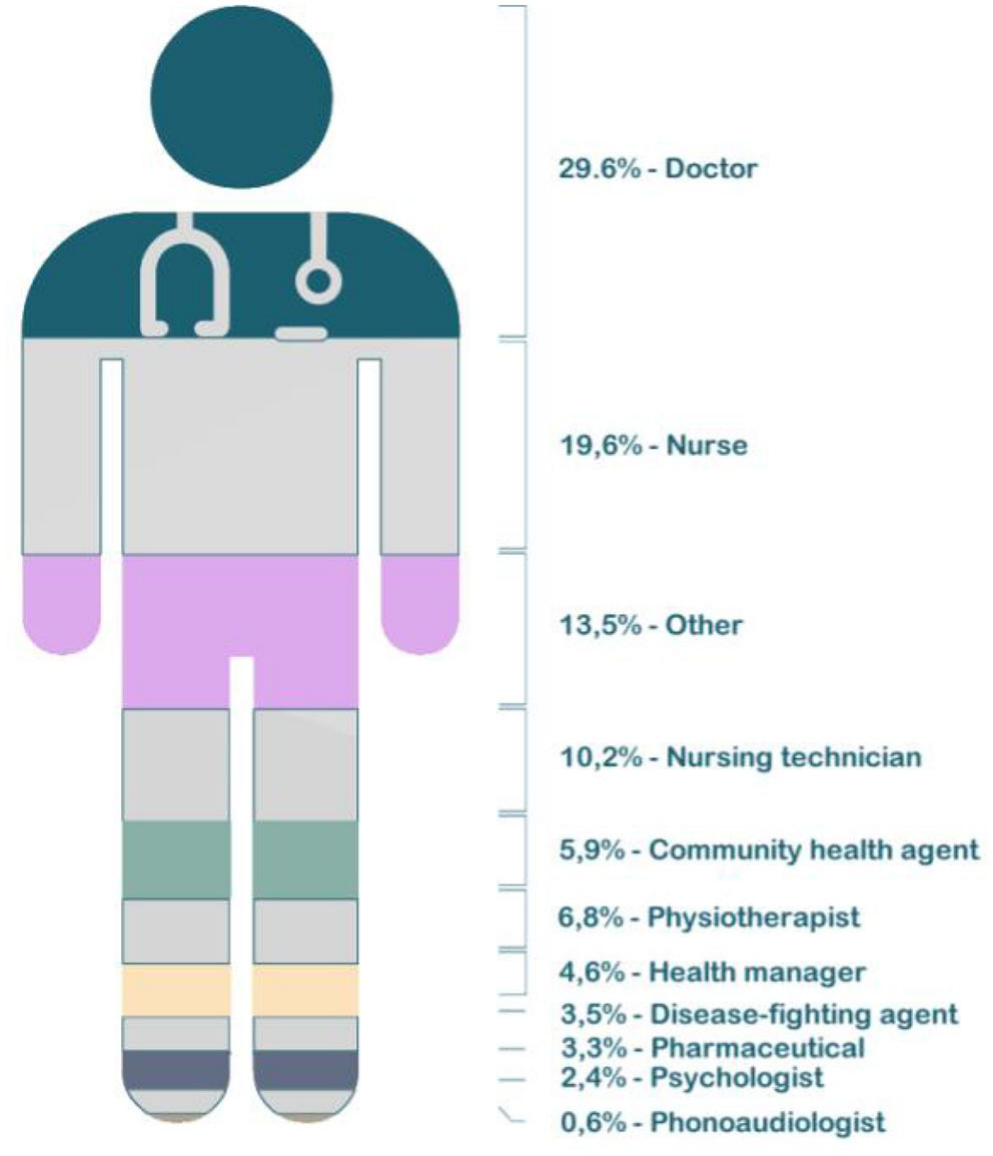
Participants by professional activity.

A total of 462 participants were healthcare workers (64.16%). Figure 1 summarizes data on course participants and the span for conducting the questionnaire. We grouped respondents' occupations, observing which professionals were the least and the most prevalent in the survey. Among a subsample of 367-course participants, there were physicians (29.6%), nurses (19.6%), nursing technicians (10.2%), physical therapists (6.8%), community health agents (5.9%), health managers (4.6%), endemic diseases combat agents (3.5%), pharmacists (3.3%), psychologists (2.4%), and speech therapists (0.6%). Figure 2 depicts the percentage of participants per occupation. All of which work in public health services in Brazil. In sum, doctors and nurses have predominantly participated in the survey. Finally, oral health assistants, nursing assistants, social workers, biomedical scientists, dentists, first responders, clinical pathology technicians, sanitary inspectors, among others, totaled 95 health workers.

Of note, participants' occupation or their purpose for enrolling in a course was not factored into the sample definition. Instead, we considered a 70% completion rate, a requirement for obtaining a certificate of achievement. All healthcare occupations mentioned above had over ten respondents. Conversely, those with ten or fewer respondents were categorized as “Other,” except for speech therapists.

**Figure 3:**
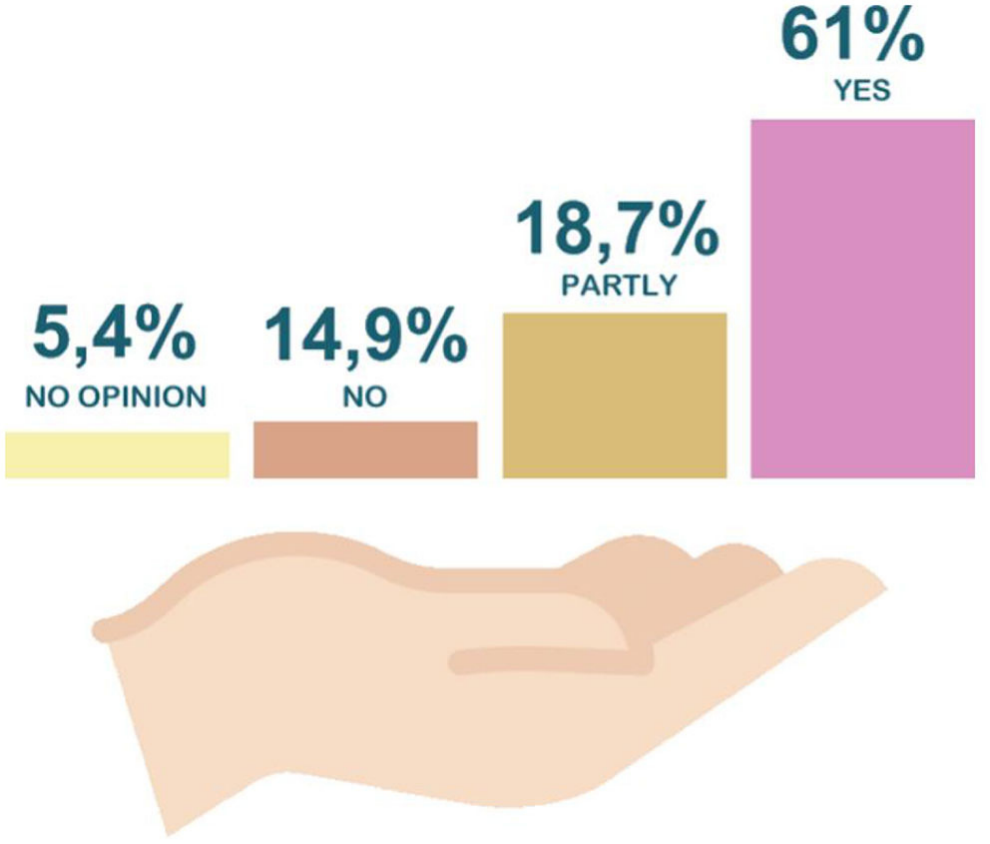
Participants by Federation Unit (FU).

**Figure 4:**
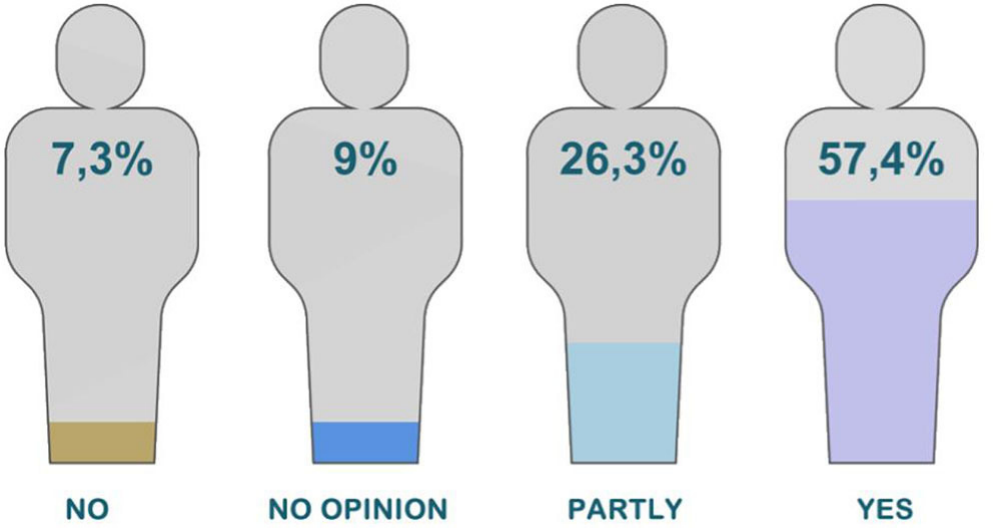
Percentage of professional establishment of participants.

When asked whether taking AVASUS courses or modules has facilitated sharing of knowledge with peers, the survey registered a 79.7% rate of affirmative answers. That mostly included physicians and nurses working in general hospitals and primary care centers. Figure 3 reveals that 61% of all participants responded “yes” and 18.7% “partly.” Moreover, when asked about the platform's contribution to enhancing teamwork, 57.4% of participants answered “yes,” and 26.3% “partly” (see Figure 4).

### 3.1. Geographic Distribution of Participants

**Figure 5:**
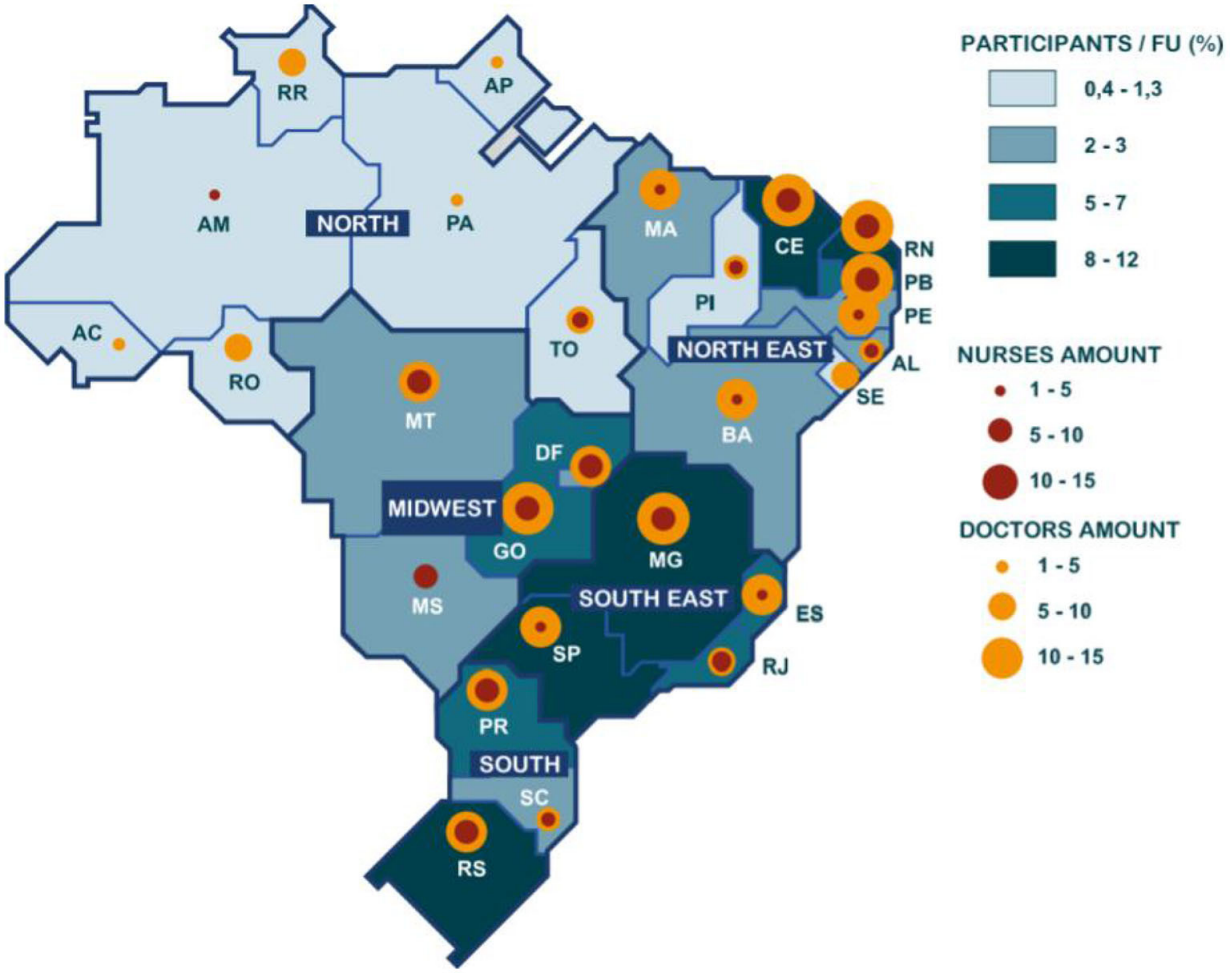
Sharing learning on AVASUS.

Considering Brazil's five major regions, participants' data show that the Northeast region had the preponderance of respondents (38.96%), followed by the Southeast (25.61%), the South (16.62%), the Midwest (13.08%), and last, the North region (5.72%). Figure 5 breaks down the spatial distribution per Brazilian region and state in relation to the health profession.

In the Northeast, physicians (36%), nurses (21%), and nursing technicians (16%) comprised the majority of health professionals participating in the survey. Interestingly, the massive participation of physicians may indicate an insufficient offer of continuing education opportunities for health practitioners from such a region. In contrast, the participation of endemic disease combat agents was quite expressive, representing 57% of the workers in this sector, nationwide, that took AVASUS courses. The state of Rio Grande do Norte (RN) had the highest number of participants in the Northeast, that is, 27% of the total. Accordingly, RN registered 38 respondents from 13 municipalities. The most significant number of health professionals answering the survey was observed in Natal (65%), the state's capital.

As for Southeast Brazil, most health professionals (40) were from Minas Gerais (MG), accounting for 43% of all respondents. The second-highest number was registered in the state São Paulo (21 participants), followed by Rio de Janeiro (18) and Espírito Santo (15). Finally, 100% of pharmacists answering the survey are also from the Southeast, expressing robust participation.

The highest density of participants in the South was from Rio Grande do Sul (RS) (46%, 28 respondents). The second-highest number of participants was registered in Paraná (25). In contrast, the lowest recorded participation was in Santa Catarina, with eight respondents. Participants from 19 municipalities of Rio Grande do Sul partook in the survey.

In the Midwest region, the largest number of answers was registered in Goiás: 19 participants from 13 different municipalities, corresponding to 40% of all participants in the region. However, no Endemic Disease Combat Agents were located in Goiás. The North Region had the smallest percentage of survey participants (5.72%). On top of that, none of them were physical therapists, endemic diseases combat agents, or psychologists. The latter profession was only registered in the Southeast.

### 3.2. Place of Work and Recommendation of Courses

**Figure 6:**
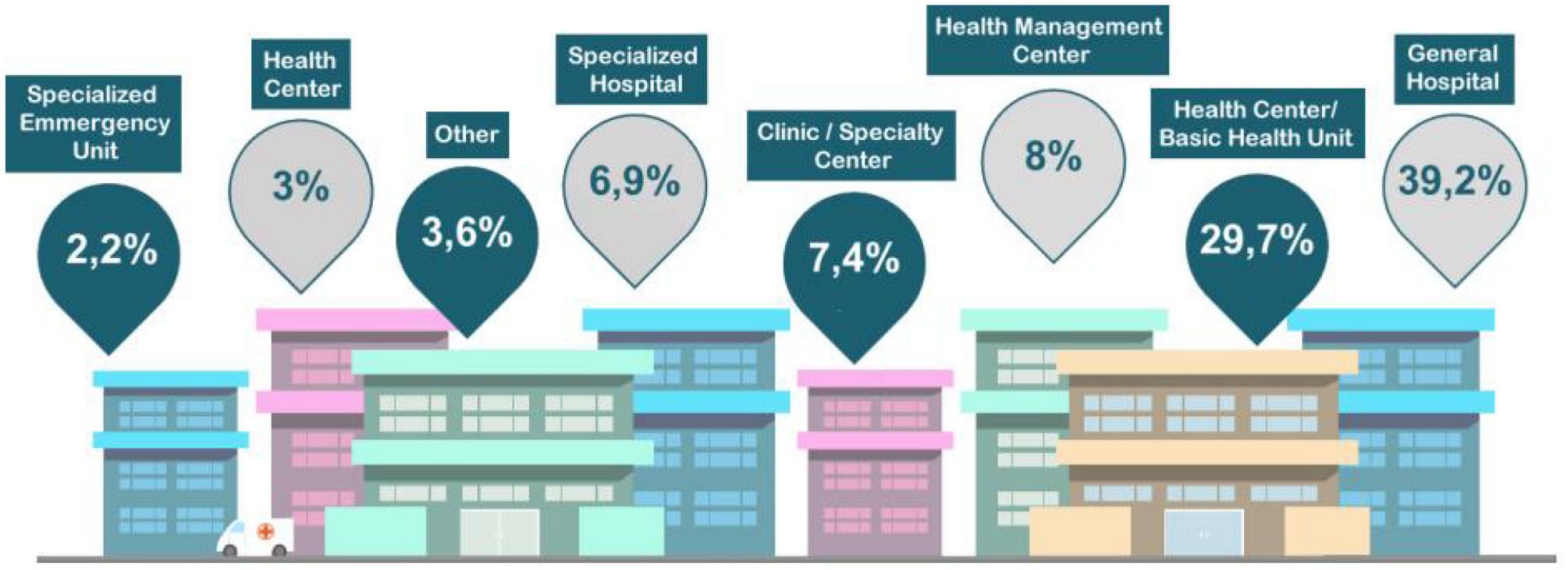
Percentage of professional establishment of participants.

Eight different categories were established. For that, information on health practitioners and their respective workplaces was crossed. The results are as follows: general hospital (32%), primary care center (32.7%), health management (8%), special care unit (7.4%), specialty hospital (6.9%), emergency care unit (2.2%), and others (3.6%). According to that distribution, there is a predominance of practitioners operating at primary care centers and general hospitals. Figure 6 details the percentage of participants per type of health facility.

**Figure 7:**
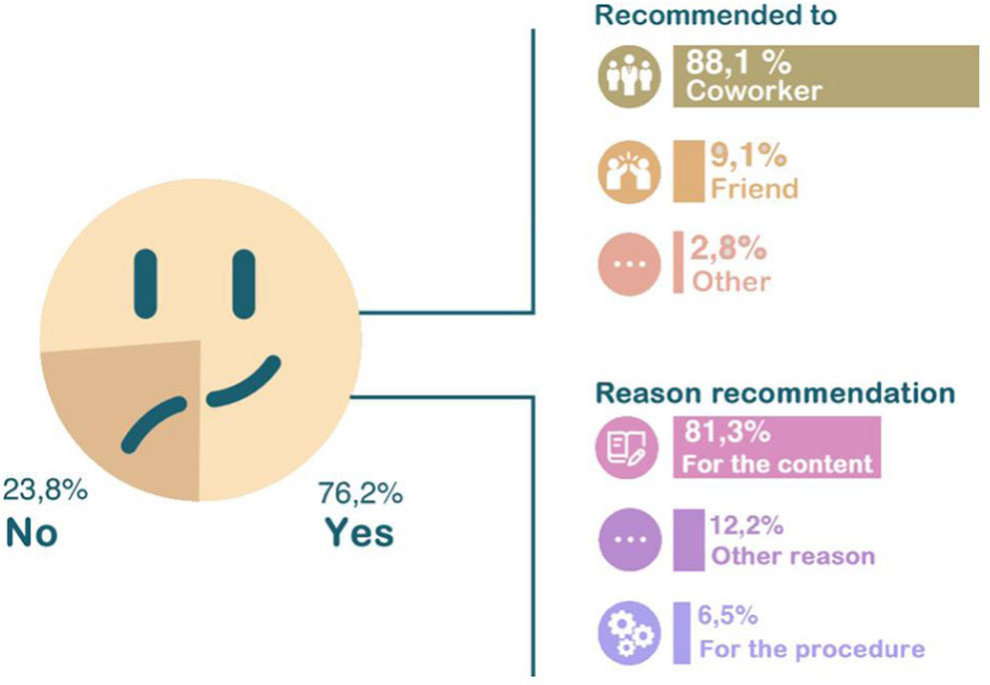
Reason for taking courses at AVASUS.

Course participants were asked whether or not they had recommended the AVASUS course in which they enrolled. As a result, 76.2% of them responded affirmatively. Such recommendations were mainly given to co-workers (88.1%) (see Figure 7).

### 3.3. Technology-Mediated Training for Meeting the Demands of Health Services

**Figure 8:**
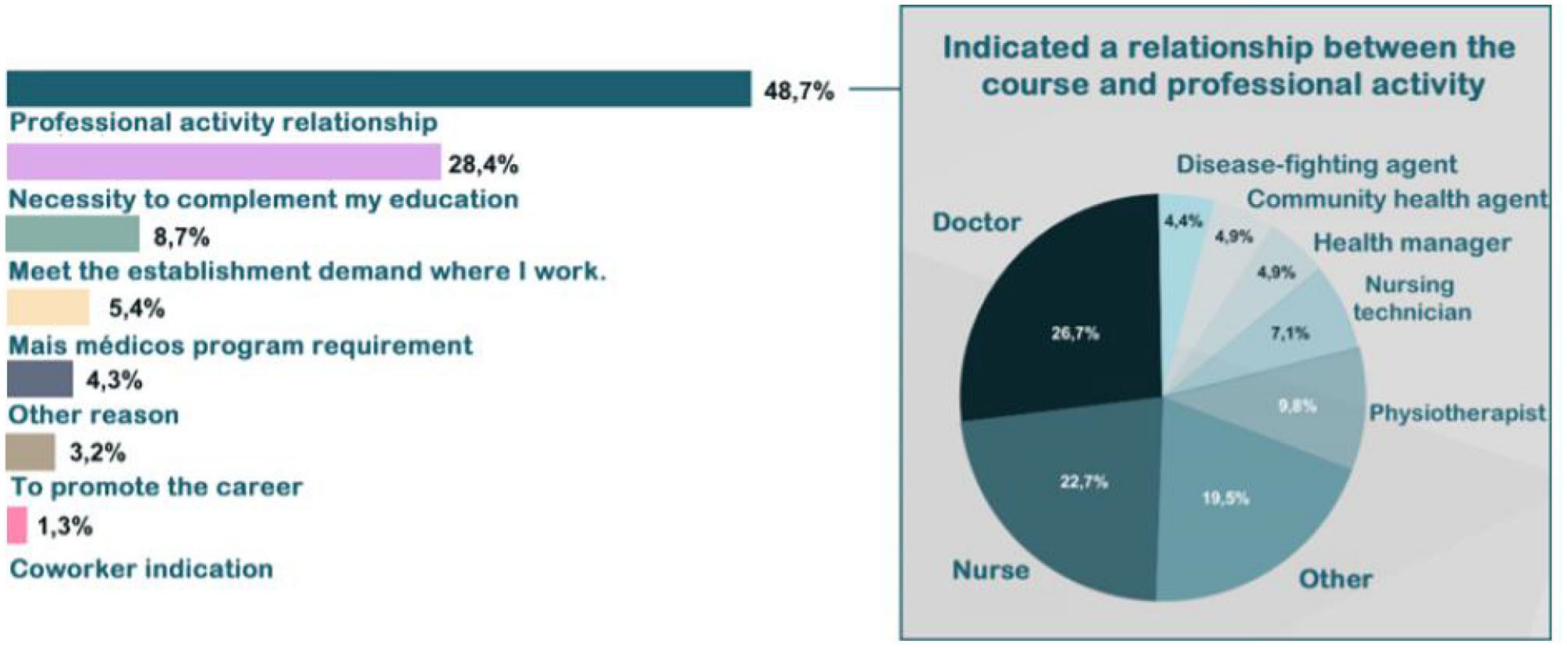
AVASUS contribution to the work.

**Figure 9:**
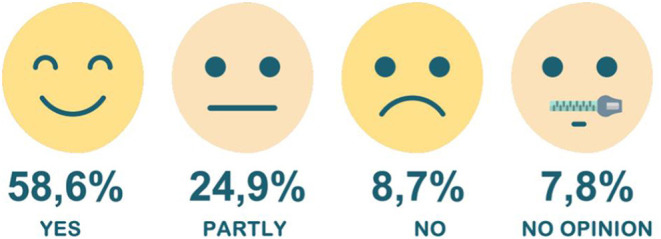
AVASUS contribution to meeting health services demands.

**Figure 10:**
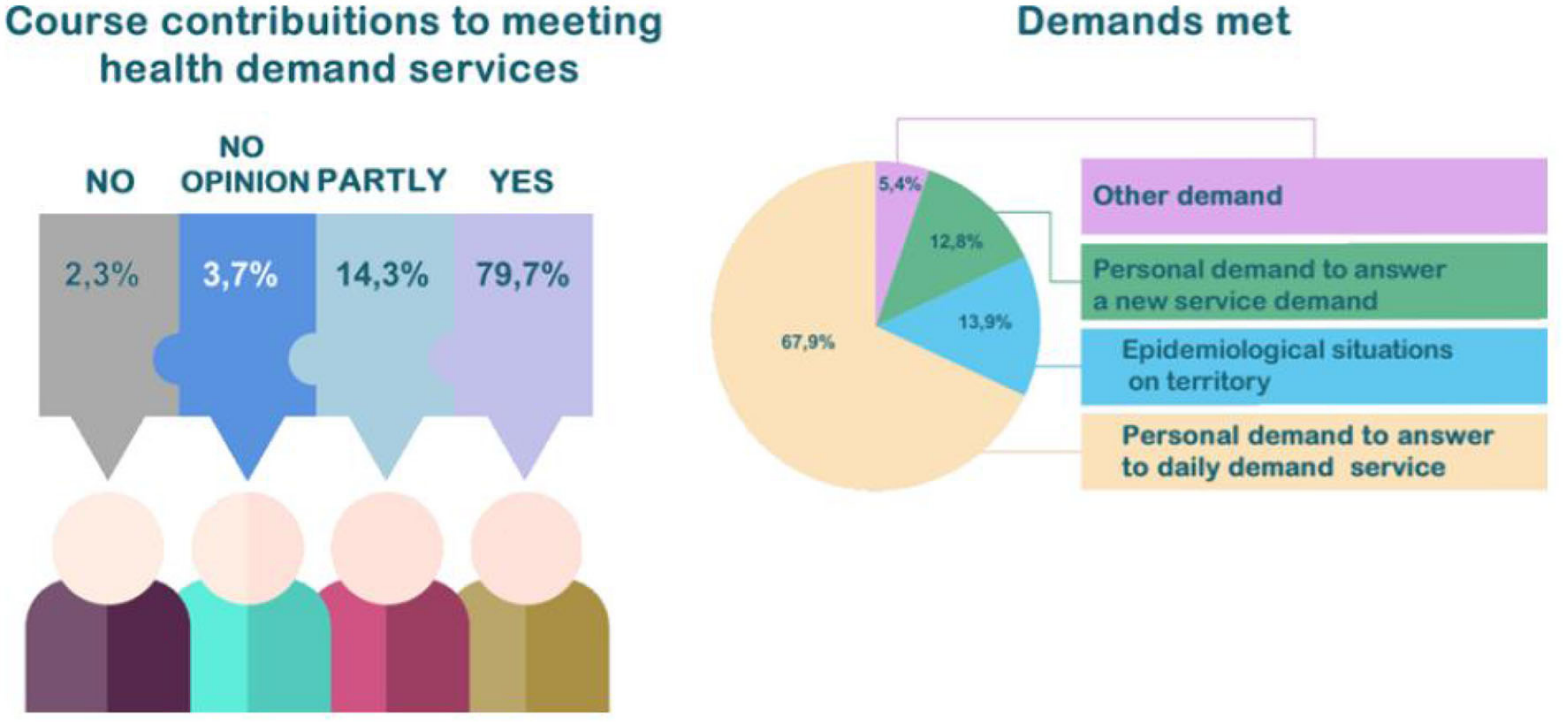
AVASUS contribution in meeting the demand of the health service.

Regarding the reason why the participants chose a specific course, the following answers and percentages were observed: relationship between participant's work and the course (48.7%); need for further health education (28.4%); meeting workplace needs (8.7%); mandatory requirement by the More Doctors Programme (25)(5.4%); job promotion (3.2%); recommendation given by co-workers (1.3%), and other reasons (4.3%). Physicians (26.7%) and nurses (22.7%) have mostly answered that they enrolled in a course due to its link to their activities. Considering the relationships among motivations and the levels of a course/module by category, the need for further health education and meeting workplace needs prevailed. Web lectures were the most sought-after course category. Health professionals who indicated the need to further their education to enroll in a course were predominantly associated with specialization and outreach categories. Figure 8 describes the reason for enrolling in AVASUS courses. Concerning whether the course/module positively impacted the work environment, in terms of decision-making, practice, or professional conduct, 58.6% responded “yes,” and 24.9% “partly” (see Figure 9). Figure 10 evidences that AVASUS made a substantial contribution to meeting the demands of health services since 94% of the answers were affirmative. Of this total, 79.7% responded “yes,” and 14.3%, “partly.” Regarding the impact on specific needs of health services, the personal demand to respond to a daily work need was deemed positive by 67.9% of respondents.

**Figure 11:**
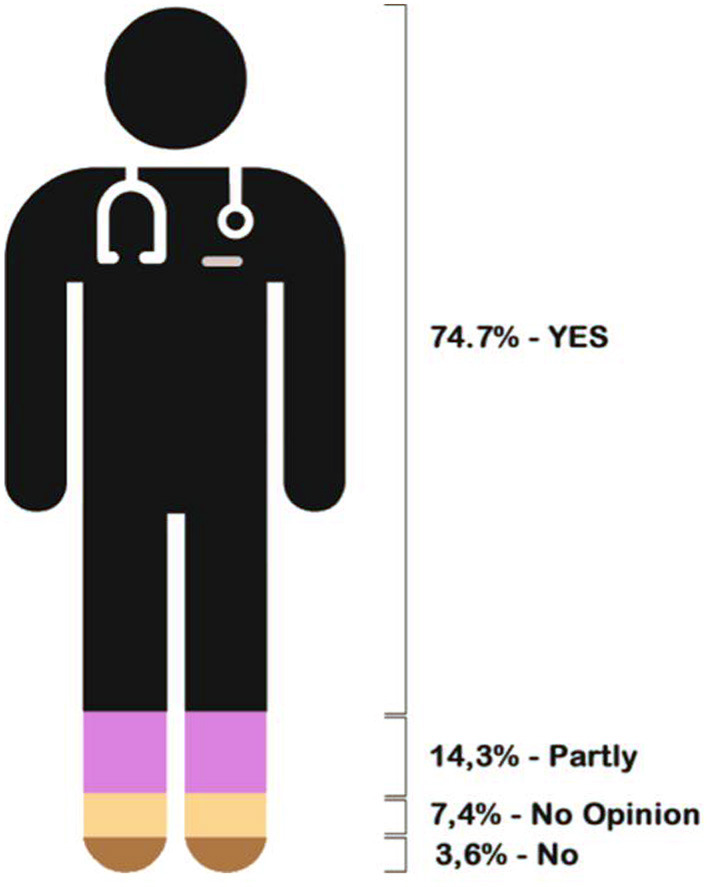
AVASUS contribution to teamwork.

“Meeting the demand of epidemiological situations in the territory” (13.9%) and “meeting new work needs” (12.8%) should also be highlighted. Knowledge acquisition, application of knowledge obtained through AVASUS courses/modules, and sharing information are considered contributions to bolster teamwork by a large share (89%) of participants. Of these, 74.7% answered “yes,” and 14.3%, “partly.” Figure 11 depicts the contribution of AVASUS to teamwork.

**Figure 12:**
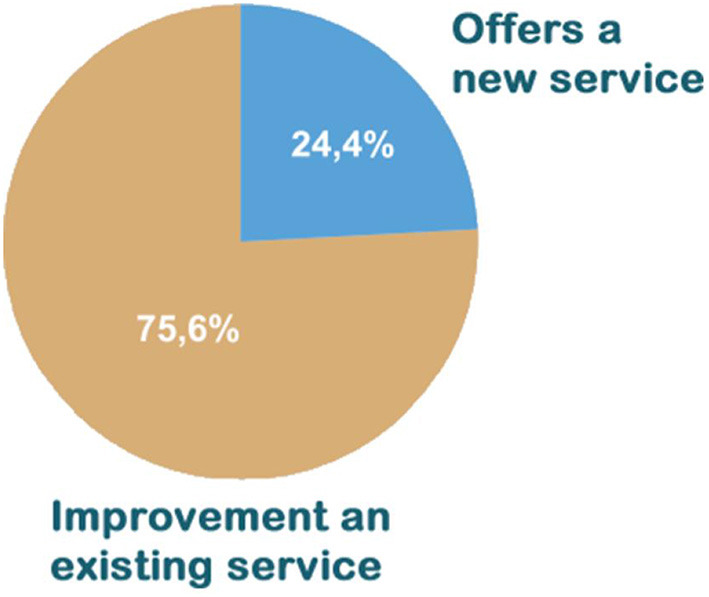
Result of the application of learning in the health establishment.

Among 60% of respondents who claimed to apply knowledge obtained in AVASUS courses in the workplace, an expressive percentage (75.6%) indicated that this application improved an existing service. Moreover, approximately 24.4% replied that they could offer a new service after the course. Figure 12 presents the pie chart with the results related to the application of knowledge obtained at the healthcare facility.

**Figure 13:**
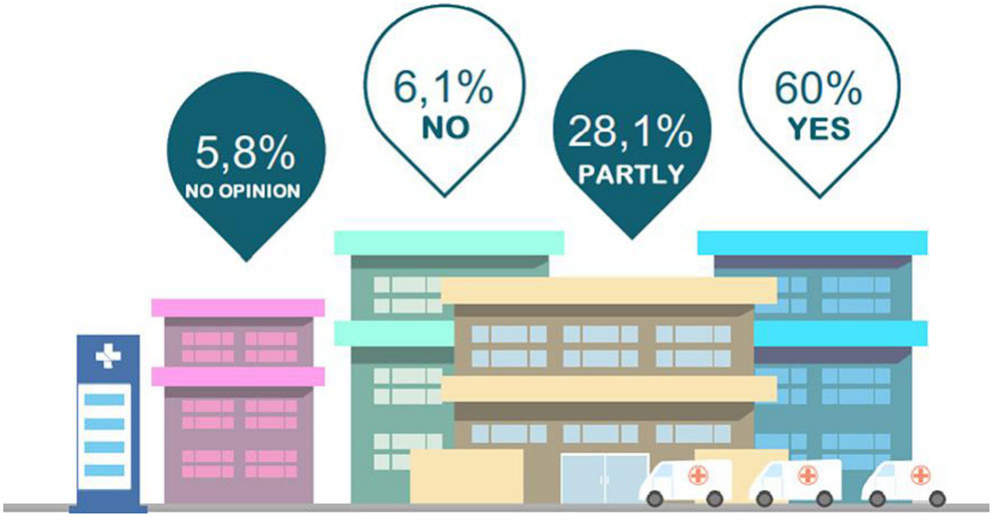
AVASUS learning application.

The application of knowledge gained from AVASUS courses/modules at the facility was pointed out by 88.1% of respondents, 60% of whom answered “yes” and 28.1%, “partly.” These figures are similar to those obtained for the three categories of courses/modules of AVASUS, as Figure 13 illustrates.

## 4. Discussion

The cornerstone of our study is the commitment to bolster the quality of healthcare training offered through AVASUS. And to enhance content and materials provided, given the educational reach of the platform. In this vein, we aimed to address and unveil the value of healthcare professionals' training and continuing education. Equally important was to reflect upon healthcare education with the latest advancements in technology into perspective. In other words, that represents an essential component of strategies to adapt and integrate emerging technologies into the education of health professionals (10). In this context, continuing education may be defined as a set of educational activities whose objective is to maintain, develop or improve the knowledge, skills and performance of working professionals–however, it differs from other training models, such as undergraduate, specialization and graduate degrees. Therefore, continuing education is an essential and strategic instrument to (i) improve the quality of health systems, (ii) improve patient health outcomes, (iii) meet health professionals needs and (iv) share and exchange knowledge (26).

Professional training is a management tool that has a strategic significance. First, it plays a critical role in innovation in education, as it favors more effective adaptation of human resources to new material resources through requalification and reconversion when necessary. Second, it provides institutions with greater flexibility when adjusting to adverse situations (10) and (26). Effectively, continuing and lifelong education for health professionals plays a pivotal role in promoting resilience in health systems, particularly during public health crises (16, 26).

Some questionnaire answers were selected for developing this article. Specifically, we grouped them based on the guiding research questions presented. Also, such data were related to pedagogical strategies, which have the problems and needs arising from the health work process and its transformation as a critical facet. A similar model was seen in (13), however, these authors only focused only on course participants' data. Conversely, in this present study, the discussion is more extensive and deeper, as the questionnaire administered also allowed the analysis of the impacts of education on work processes in health services in Brazil.

The first guiding question focused on a pivotal aspect of meaningful learning. In this era of information sharing, skills that trigger this sort of learning are necessary. In this context, 61% of respondents indicated sharing the knowledge gained through AVASUS courses/modules. This result unveils advances and changes that can bolster new ways of thinking and building knowledge. The answers about AVASUS's contributions to improving teamwork corroborate this result, considering that sharing knowledge favors dialogue and ideas among peers. Thus, respondents pointed out that the platform's courses have thoroughly (57.4%) or partly (26.3%) strengthened teamwork. Thus, it may suggest that this training contributed positively to the work environment. Therefore, it can be ascertained that taking the AVASUS course/module also was important to improving the health service provision.

The final verification on the positive impact of AVASUS was corroborated through the question about the recommendation of courses. This aspect can be considered a quality indicator–a set of signs that readily qualify the training activities carried out on the platform. In this context, 76.2% of the answers were positive for recommending the courses or modules. Therefore, when explaining the reason for suggesting the platform offerings, the quality of the courses/modules taken was the prevailing factor. Hence, these results justify the broad coverage of AVASUS as to health professional education and the positive repercussions it has caused in health service providers' training.

The second guiding question addressed the compatibility among AVASUS' educational offerings and what healthcare services and systems demand, which is another essential quality indicator. The reason for the courses' participants to seek courses and modules was investigated. It is known that motivation is fundamental for enrolling and following through with a course. Aside from that, it mainly involves goal orientation and its relation between intrinsic motivation, which is internal to the individual. Thus, being profound and long-lasting, and the extrinsic motivation arising from environmental stimuli.

It was found that the primary motivation was intrinsic: the link between the course and the work developed (48.7%), in addition to the need for further professional training (28.4%). Only then does an extrinsic motivation emerge, which was meeting the demand of the health establishment where the course participant is employed (8.7% of responses). Hence, this is considered a positive result.

Still, within the scope of this topic, the contributions of activities carried out at the health service were verified, which are significant in the relationship between theory and practice. So, 79.7% of the “yes” answers were verified, which expresses total meeting of the demands. Another 14.3% of participants responded “partly.” Thus, it can be noted that 94% of the answers indicated an agreement between health services needs and the topics covered in AVASUS. This high rate of positive responses stands out in a scenario of health services provision, especially when considering the epidemiological issues that repeatedly occur in the country, such as the arboviruses (Zika, Dengue, and Chikungunya).

Another question is, constant attention has been given to how courses contribute to teamwork improvement and how the change in work processes affects the quality of health services. That is also important for improving the educational offerings made available on the platform. This improvement does not only involve acquiring course materials, the development of new intellectual and attitudinal competencies, and enhancing professional practices. Equally important is the cultivation of interaction between peers, the ability to understand and coexist within distinct points of view, and the development of critical judgment about knowledge. Therefore, the majority of respondents indicated “yes” (74.7%) and “partly” (14.3%) to the aspect under discussion. It is indeed encouraging to find that healthcare workers have been sharing what they have learned through AVASUS. Mainly when its impact on healthcare education is being assessed and reflected upon. When a student acts as an agent for knowledge dissemination, future costs lessen, and optimized training improves, in this particular case, the provision of health services. Besides, such exchange occurs on a large scale. Thus, besides amplifying practitioners' impact on health services, sharing knowledge is crucial for it may lead to investments for training them more efficiently.

The third guiding question is directed toward the future and is associated with innovation. It was detected that 60% of course participants applied the knowledge obtained through AVASUS courses within their work environment. Hence, improving what already exists and works suitably can be considered the first stage of innovation, which some authors call incremental innovation, allowing renewal of teamwork and professional practice. In sum, 75.6% of responses in this field pointed to the fact that the content of the courses/modules contributes to the improvement of existing health services. Approximately 25% of the sample (precisely, 24.4%) mentioned that the content learned could trigger the offer of new services or showed a completely different way of doing something that was done in a restricted way, which is the so-called radical or disruptive innovation.

From the results obtained, it can be said that the impacts of the courses available in the platform are (1) positively evaluated by course participants and (2) promote meaningful, immersive, and collaborative learning that is compatible with the needs of healthcare services. Beyond that, they also enable reflections about current practices, stimulate the development of new forms of working, offer new services, and adopt technologies while promoting the improvement of existing services and fomenting innovation.

According to Allen et al. (27) health impact assessment should measure beyond course outcomes in relation to the quality of education. Therefore, it is necessary to understand and evaluate the context. Following the idea proposed by Allen et al. (27), when also observing “how and why the program worked and what else happened”, it is possible to understand that the educational offers implemented in AVASUS not only promoted an increase in continuing education in health. By analyzing the context of educational interventions in the Brazilian Health System (SUS), it was possible to verify the effectiveness in inducing the health system's responses in public health crises. For example, in COVID-19, AVASUS acted as an important tool for inducing resilience, as well as in the syphilis epidemic (16), where Brazil, after starting the process of massive training at AVASUS (15), began to register in the same period an increase in testing and the consequent reduction in cases of congenital syphilis (20–23).

## 5. Conclusion

Access to health care is not necessarily contingent on health workforce availability. Guaranteeing the continuing education of professionals operating from urban centers to the most remote areas is, in effect, strategic for delivering quality and equitable access to health care to all SUS users. Indeed, the scalability of education for a health workforce numbering millions represents a challenge that can be overcome through digital technological mediation, with educational offerings designed to tackle epidemiological challenges in the territories and at each specific moment.

This study reports data and results of educational offerings registered in AVASUS, which point to the adequacy of the massive, open, flexible, and digital-technologically mediated learning strategies designed to contribute to the Sustainable Development Goals (SDGs) set forth by the UN in the 2030 Agenda, specifically, SGD 3 and 4 (28).

Furthermore, the digital age provides socioeconomic and cultural opportunities in which knowledge and learning are turned into valuable assets for each individual. Accordingly, the results of this research, whose objective covered the AVASUS courses, reveal the positive impacts of the continuing health education offered to the SUS workforce and health students.

The significance of our results and analyses comes from their consistency with the health services needs, including those that emerged during major sanitary crises in Brazil. In addition, these analyses pointed out that the courses contributed to stimulating the development of novel working practices, thus leading to changes in the work processes. According to the data presented, this factor has led to impacts that improved Brazilian health and positively contributed to the development of new health services, as emphasized in the results and discussion section.

In addition, the technological mediation offered by Massive Online Open Courses (MOOCs) in AVASUS has spurred the adoption of innovative technologies in the continuing process of improving the individual and collective capacities of the Brazilian health workforce.

Considering the triangulation of aspects addressed in this paper, the effective sharing of knowledge attained through the AVASUS courses in the workplace demonstrated that they meet, strengthen, and enhance the needs of health education and service offerings. This is the reason for this paper's title, which refers to the alignment of AVASUS with the complex needs of the health system in a country of a continental size as Brazil.

In this context, AVASUS has become a remarkably efficient and effective tool for promoting public health policies. Its educational offerings have well-trained human resources in health and added value to improve health care for Brazilian citizens. Based on this fact, AVASUS represents an experience that can be replicated in other countries with similar aspects.

From the standpoint of AVASUS contributions as educational technology, this research also allowed us to observe the relevance of massive health education. In other words, how technological mediation escalated this process, especially in situations of health crises and emergencies.

In April 2022, AVASUS surpassed the mark of 2,2 million enrollments in more than 320 online educational offerings. Between 2015 and 2022, AVASUS had prominence in at least three public health emergencies in Brazil. Therefore, as an unfolding event and from a perspective of adequacy and constant improvement of educational offerings, it is substantial to consider studies and analyses that can assess the impacts of massive training for the SUS workforce, as follows:

- Examine the impacts and contributions, not only on health services but on the health system comprehensively;
- Evaluate the results of massive training to address healthcare needs and public health emergencies, and
- Analyze the relevance of technology-mediated continuing education activities to assess the promotion of SUS resilience and responsiveness.

Considering future possibilities and the study presented in this paper, further research could explore to what extent massive, open, flexible, and digitally mediated health education can impact daily and community life, thus promoting equity and mitigating local and regional inequalities in Brazil.

## Data Availability Statement

The analyzes for this work were made with public data available on AVASUS at the following link: https://zenodo.org/record/5888398#.YerUIhPP3Fo.

## Author Contributions

RV and DB: organizing and manuscript review. SC, ER, and IM: performed the analysis of the data. RV, EO, DB, and ER: preparing the manuscript. CO, SC, CG, AC-O, KC, and RC: manuscript review and modification. All authors editing, revision, read, and approved the final manuscript.

## Funding

Fundação Norte-Rio-Grandense de Pesquisa e Cultura, Universidade Federal do Rio Grande do Norte.

## Conflict of Interest

The authors declare that the research was conducted in the absence of any commercial or financial relationships that could be construed as a potential conflict of interest.

## Publisher's Note

All claims expressed in this article are solely those of the authors and do not necessarily represent those of their affiliated organizations, or those of the publisher, the editors and the reviewers. Any product that may be evaluated in this article, or claim that may be made by its manufacturer, is not guaranteed or endorsed by the publisher.
